# The role of ICAM-2 in neuroblastoma

**DOI:** 10.18632/oncoscience.273

**Published:** 2015-11-23

**Authors:** Karina J. Yoon, Aubrey L. Miller, Kelly M. Kreitzburg

**Affiliations:** Department of Pharmacology and Toxicology, University of Alabama at Birmingham, Birmingham, AL, USA

**Keywords:** intercellular adhesion molecule-2, neuroblastoma, metastasis

The role of immunoglobulin superfamily cell adhesion molecules (CAMs) in facilitating immune responses in normal and tumor cells is well established. Cell adhesion molecule-1 (CADM1), for example, suppresses development of mouse mammary tumor cell metastasis by interacting with CD8^+^ T cells in immune-competent hosts [1]. Similarly, co-expression of Intercellular Adhesion Molecule-2 (ICAM-2) and chemokine C-X-C motif ligand 17 (CXCL17) elicits anti-tumor immune responses and suppresses tumor growth [2]. Although controversial, current literature suggests that proteins of the immunoglobulin superfamily CAMs also have functions that may well be distinct from their roles in mediating immune responses. Junctional adhesion molecule-A (JAM-A), for example, negatively regulates breast cancer cell invasiveness by disrupting tight junctions [3]; and a member of the B7 family of the immunoglobulin superfamily proteins, B7-H3, impairs osteogenic differentiation *in vitro* and *in vivo* [4].

Our lab demonstrated that ICAM-2 inhibited the development of disseminated neuroblastoma tumors in a preclinical model of metastatic neuroblastoma [5-7]. This inhibition depended on the interaction of ICAM-2 with the actin cytoskeletal linker protein α-actinin, an interaction that inhibited cell motility [7]. Ectopic expression of ICAM-2 did not affect the tumorigenic potential of neuroblastoma cells [7]. Importantly, immunohistochemical analyses of primary neuroblastoma tumor specimens demonstrated that neuroblastoma cells expressing ICAM-2 are phenotypically and histologically those recognized clinically to have limited metastatic potential [5]. Since metastatic disease is responsible for >90% of cancer-related deaths for multiple types of solid tumors, we suggest that elucidation of the molecular mechanism by which ICAM-2 suppresses the metastatic potential of neuroblastoma cells would identify proteins or pathways that might be exploited therapeutically to prevent metastatic disease progression.

In normal tissues ICAM-2 is expressed predominantly by neovascular endothelial cells, and at lower levels by established vasculature and some leukocytes. The 202 amino acids comprising its extracellular domain mediate binding of ICAM-2 on endothelial cells to β_2_-integrins on the surface of leukocytes, to facilitate migration of neutrophils through the vascular endothelium as a component of immune reactions [8]. In neuroblastoma cells ICAM-2 inhibits cell motility independent of immune response, as we observed this inhibition in wound healing and modified Boyden chamber assays *in vitro*, assays that clearly lack an immune component [5,7].

*In silico* modeling indicated that ICAM-2 with mutations in the proposed α-actinin binding domain had a more ‘closed’ configuration than the wild type protein, and predicted that these ICAM-2 mutants would not interact with α-actinin [7]. Co-immunoprecipitation experiments confirmed *in silico* predictions [7]. In support of these findings, the interaction of ICAM-2 with α-actinin was essential to inhibit development of disseminated tumors *in vivo* [7]. As mentioned, ICAM-2 did not affect tumorigenic potential, and unpublished data showed no effect of ICAM-2 on expression of epithelial-mesenchymal transition (EMT) or stemness markers.

Although we regard the inhibition of tumor cell motility by ICAM-2 *in vitro* to be compelling evidence that ICAM-2 participates in cell functions distinct from immune responses, we acknowledge the complex nature of metastatic tumor progression and propose that our data suggest multiple hypotheses with respect to the mechanism by which ICAM-2-inhibits neuroblastoma cell motility. The first hypothesis is that the intracellular interaction of ICAM-2 with α-actinin initiates ‘inside out’ signaling, and causes conformational changes in the extracellular domain of ICAM-2 that facilitate (or inhibit) interactions with specific extracellular matrix proteins that play an integral role in cell motility. The second is that the interaction of ICAM-2 with α-actinin alters the conformation of α-actinin to facilitate (or inhibit) the association of this actin cytoskeletal protein with alternative binding partners, with ICAM-2 acting as an ‘activator’ (or inhibitor) of α-actinin rather than as a membrane anchor protein. The third hypothesis, based on unpublished microarray data, is that ICAM-2 expression indirectly up-regulates protein tyrosine phosphatases (PTPs) involved in the formation and maintenance of focal adhesions such as focal adhesion kinase (FAK), Src, or Rac and Rho GTPases, each of which has crucial role in tumor cell motility.

Our data show that ICAM-2 inhibits tumor cell motility and suppresses the metastatic potential of neuroblastoma cells. We propose that elucidating molecular events associated with ICAM-2 expression will identify key protein interactions that regulate the metastatic process in this cell type.
